# Periscope: Parturiology

**Published:** 1818-07-01

**Authors:** 


### PARTURIOLOGY.

Although spontaneous evolution of the fœtus often takes place, and would perhaps take place still oftener than it does, were the natural efforts trusted to; yet we can seldom venture to place so much confidence in the powers of nature. The operation of turning is a very laborious and a very difficult measure, in the majority of cases where it is necessary, and therefore any rule which can facilitate the manœuvre is highly deserving the attention of the public.

Dr. Brien, of Dublin [*Ed. Journ.* 53.] lays it down as a general regulation, that, in turning the child, the operator should not remove the inferior extremities from their natural position, provided he can effect his purpose while they remain in it; and this, he thinks, can be done in most instances. There are, he observes, but few cases where it is necessary to proceed to this operation before the os uteri is dilated; and this being favourable, the patient is to be placed on her left side; and the *left* hand of the surgeon, lubricated, is to be slowly and cautiously introduced, conically, through the vagina and os uteri, along the abdomen of the child, on which, as much as can be, it is to lie at rest during each pain. In the intervals of pain, the hand must be pushed upwards until it arrive at one of the knees; one or two fingers should now be hooked in the flexure of this part. The operator must then draw the knee downwards and forward, towards the centre of the great diameter of the brim of the pelvis, and, if any difficulty occur, he will, at the same time, endeavour gently to push up the presenting part. Should the child still continue jammed, after using moderate force, he recommends the situation of the hand in utero to be varied, and the fingers to be hooked in the flexure of the other knee. When by this procedure one foot is brought within the reach of the noose, it may be sometimes necessary, after applying one, to retrace the same steps to bring the other within the power of a second application.

